# Signalling Network Construction for Modelling Plant Defence Response

**DOI:** 10.1371/journal.pone.0051822

**Published:** 2012-12-18

**Authors:** Dragana Miljkovic, Tjaša Stare, Igor Mozetič, Vid Podpečan, Marko Petek, Kamil Witek, Marina Dermastia, Nada Lavrač, Kristina Gruden

**Affiliations:** 1 Department of Knowledge Technologies, Jožef Stefan Institute, Ljubljana, Slovenia; 2 Jožef Stefan International Postgraduate School, Ljubljana, Slovenia; 3 School of Engineering and Management, University of Nova Gorica, Nova Gorica, Slovenia; 4 Department of Biotechnology and Systems Biology, National Institute of Biology, Ljubljana, Slovenia; 5 Institute of Biochemistry and Biophysics, Polish Academy of Sciences, Warsaw, Poland; 6 The Sainsbury Laboratory, Norwich Research Park, Norwich, United Kingdom; Centro de Investigación y de Estudios Avanzados, Mexico

## Abstract

Plant defence signalling response against various pathogens, including viruses, is a complex phenomenon. In resistant interaction a plant cell perceives the pathogen signal, transduces it within the cell and performs a reprogramming of the cell metabolism leading to the pathogen replication arrest. This work focuses on signalling pathways crucial for the plant defence response, i.e., the salicylic acid, jasmonic acid and ethylene signal transduction pathways, in the *Arabidopsis thaliana* model plant. The initial signalling network topology was constructed manually by defining the representation formalism, encoding the information from public databases and literature, and composing a pathway diagram. The manually constructed network structure consists of 175 components and 387 reactions. In order to complement the network topology with possibly missing relations, a new approach to automated information extraction from biological literature was developed. This approach, named Bio3graph, allows for automated extraction of biological relations from the literature, resulting in a set of *(component1, reaction, component2)* triplets and composing a graph structure which can be visualised, compared to the manually constructed topology and examined by the experts. Using a plant defence response vocabulary of components and reaction types, Bio3graph was applied to a set of 9,586 relevant full text articles, resulting in 137 newly detected reactions between the components. Finally, the manually constructed topology and the new reactions were merged to form a network structure consisting of 175 components and 524 reactions. The resulting pathway diagram of plant defence signalling represents a valuable source for further computational modelling and interpretation of omics data. The developed Bio3graph approach, implemented as an executable language processing and graph visualisation workflow, is publically available at http://ropot.ijs.si/bio3graph/and can be utilised for modelling other biological systems, given that an adequate vocabulary is provided.

## Introduction

Plants and pathogens can enter into various relations that do not necessarily damage the host plant. In resistant interaction the plant cell efficiently perceives the pathogen signals, often through the interaction between the resistance (R) protein and the pathogen avirulence factor [1]. This interaction initiates a complex signalling network, referred to as *plant defence response or plant defence signalling* (PDS), orchestrating the activity of a multitude of transcriptional regulators, resulting in massive changes of the gene activity and extensive reprogramming of the cell metabolism. For a successful defence the activation of plant response must be rapid, efficient and targeted [2]. It was shown that salicylic acid (SA), jasmonic acid (JA) and ethylene (ET) play a crucial role in mediating the defence signalling responses in plants [3]. SA is generally involved in the activation of the defence responses against biotrophic and hemi-biotrophic pathogens, whereas pathogens that kill the host and feed on the contents (necrotrophs) and herbivorous insects are generally affected by JA/ET-mediated defences [4]–[5]. Although most reports indicate an antagonistic interaction between SA- and JA-dependent signalling, synergistic interactions have been described as well [6]–[8]. ET often plays a modulating role [9]–[11]. The interconnected PDS network provides plants with an enormous regulatory potential to rapidly adapt to their biotic environment and to utilise their limited resources for growth and survival in a cost-efficient manner [12]. This is especially important when the plant is exposed to multiple attackers [13].

Systems biology was proven to be successful in modelling complex biological processes [14]. Before the analysis of the network dynamics, one needs to understand the network structure [15]. There are various representation formalisms that can be used to represent a network topology, including the directed graphs formalism as used in the Systems Biology Graphical Notation by Le Novère et al. [16] or the modified EPN (mEPN) scheme proposed by Raza et al. [17]. To construct the signalling network topology, different information sources can be used, including pathway databases such as the KEGG Pathway [18], Reactome [19] and BioCyc [20], integrated knowledge sources such as ONDEX [21] and Biomine [22]–[23], and the scientific literature itself. Given that most of human biological knowledge is still stored only in the silos of biological literature, retrieving information from the literature is required when building the signalling network topology.

Scientific literature can be inspected manually or analysed by natural language processing and information extraction tools. There are numerous biological models which were manually constructed based on an in-depth literature survey, such as the macrophage activation model developed by Raza et al. [17] [24]. On the other hand, state-of-the-art technologies enable information extraction from scientific texts in an automated way by means of text processing techniques, based on the advances in the area of natural language processing (NLP) of biology texts (see e.g., the research advances of the emerging bioNLP community at http://www.bionlp.org/). Similar to our work which involves the extraction of a set of *(component1, reaction, component2)* triplets from biology texts, several existing NLP tools enable the extraction of interactions between the components (e.g., see the review by Ananiadou et al. [25]). The most common NLP approaches can be classified into three categories [26]: rule-based approaches, machine-learning approaches and co-occurrence-based approaches. Examples of rule-based systems include GeneWays [27], Chilibot [28], PLAN2L [29] and the approach proposed by Ono et al. [30]. Combined methods, including co-occurrence-based approaches, such as the one developed by Suiseki et al. [31] and upgraded in the BioRAT system by Corney et al. [32], are less appropriate for systems biology as the information retrieved is partial and can therefore not be directly transformed into a graph format used for signalling network modelling. In most systems, the information is retrieved only from abstracts; an exception is BioRAT which can process full texts, albeit using a quite general vocabulary [32].

Most of the above mentioned approaches enable the users to query the extracted information, but do not result in an explicit network topology which can be visualised for simple inspection by the biology experts. Exceptions are the Chilibot system by Chen and Sharp [28], the approach by Blaschke et al. [33] and the GeneWays system by Rzhetsky et al. [27]. These systems are however not directly applicable in our context for the following reasons. The Chillibot system enables the search for relations by querying only a limited number of entities without supporting the complete network topology construction. Blaschke et al. [33] extract information only from abstracts. The closest to our work is the GeneWays system [27] which enables the extraction, analysis, visualisation and integration of molecular pathway data, but the system is not publicly available.

When studying plant-pathogen interactions most of the research has focused on individual interactions or subsets of the whole PDS mechanism [34]–[35]. The first attempt to model PDS by constructing a small Boolean network and performing numerical simulations of PDS was proposed by Genoud et al. [36]. However, this model is simple, containing 18 biological entities and 12 Boolean operators, whereas to fully describe complex biological systems one needs to simultaneously address a large number of components [14].

The goal of this work is to construct a topology of the PDS model aimed to improve our understanding of this complex signalling system and serve as a basis for dynamic modelling and simulations. The dynamic PDS model can be useful in predicting plant behaviour under conditions that have not yet been tested experimentally. The intended use of the model is to generate predictions of dynamic behaviour of selected variables by model simulation under different conditions and to use the simulation results to suggest new experiments. Simulations are faster than the laboratory experiments that involve a time-consuming process of creating mutant plants with modified gene expressions in a particular pathway. Therefore, simulation results can help to identify the key components for gene mutations in the PDS and assist in further experimental research. Such an approach to experiment design is considered more helpful than a simple intuitive or experimental trial and error approach. In this study we concentrated on model plant species *Arabidopsis thaliana* and its interaction with viruses. At the level of signal perception we focused on the Turnip Crinkle Virus (TCV) infection, while the remaining PDS network was built from the available knowledge on SA, JA and ET signalling and the crosstalk between these pathways.

In our work, the PDS network topology was initially manually constructed by defining the representation formalism, the vocabulary of components and relations, as well as the actual signalling network topology. Nevertheless, we later realised that this time-consuming process should be best complemented by automated triplet extraction from literature and network composition from the extracted triplets of the form *(component1, reaction, component2).* In addition to building this model topology, our motivation was also to build a tool which can be utilised for automated model topology construction in other domains.

Given that this work is relevant to scientists from different fields (biology and computer science), Table 1 introduces the mapping of the terms used in these two areas.

**Table 1:** Mapping between biology and computer science terminology used in this paper.

Biology	Computer science
Biological system	Computer model, Network
Pathway	Sub-model, Sub-network
Molecule, Component	Node, Vertex
Reaction, Interaction	Relation, Edge, Arc, Link

## Results and Discussion

The topology of the PDS model, which is the final outcome of this paper, was constructed in three steps: manual construction of the PDS model topology, automated extraction of the network structure from biological literature, followed by expert curation and automated merging of these two networks. To efficiently implement the second step we developed a new methodology and a tool, named Bio3graph. The Bio3graph methodology is implemented as a reusable workflow of natural language processing components for information extraction from biological literature in a format compatible with systems biology formalisms, and workflow components for graph construction and visualisation.

### Manually Constructed PDS Model Topology

When constructing the PDS model topology, we focused on metabolic, signalling and gene regulation networks of SA, JA and ET, as they have a crucial role in mediating the induced defence responses in plants [3]. When identifying the relevant biological reaction and component types, we followed the mEPN formalism [17], slightly adapted to our requirements.

**Figure 1:**
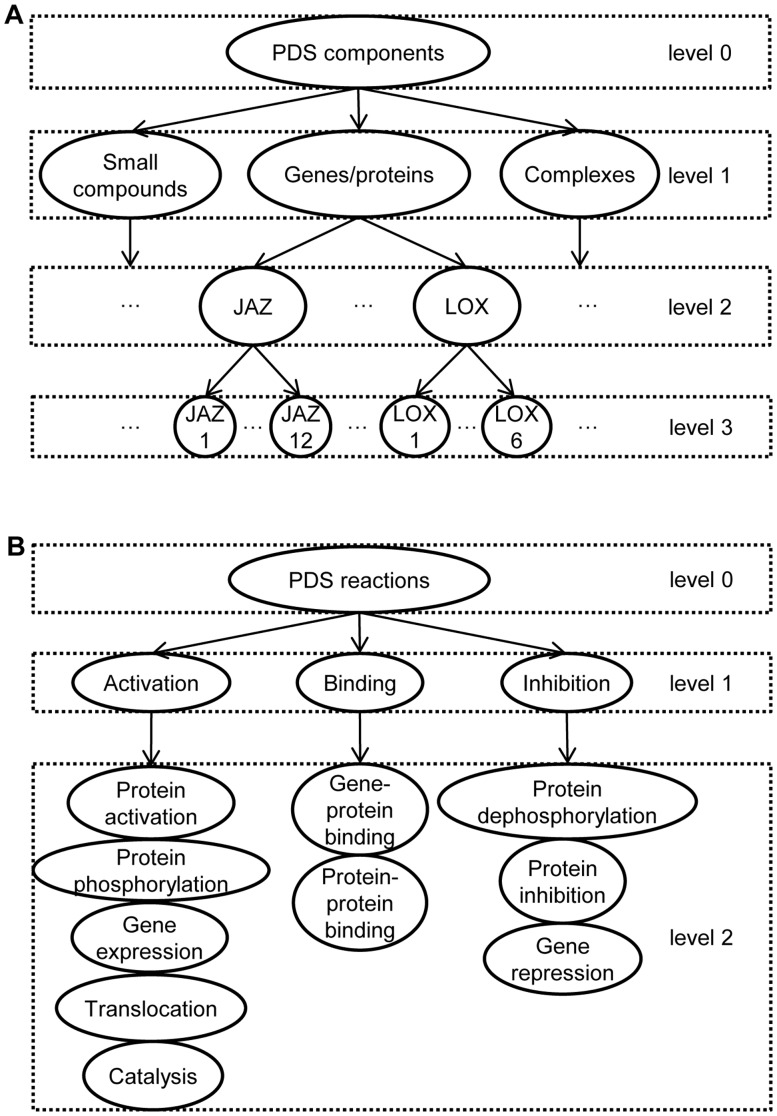
Taxonomy of PDS components and reactions. A) In the taxonomy of PDS components there are four representation levels. The highest level (level 0) is the most abstract level, while the lowest one (level 3) represents single molecules. B) In the taxonomy of PDS reactions individual reactions are represented at the lowest level (level 2) and are grouped according to their functionality into three groups at level 1: *Activation (A)*, *Binding (B)* and *Inhibition (I)*.

The following component and reaction types were identified. Three groups of biological components were considered (see Figure 1A): small compounds or metabolites (chorismate, jasmonic acid, linoleic acid, etc.), genes/proteins (chorismate synthase, PAD4, etc.) and complexes (JA-Ile/COI1/SCF complex, etc.). The following reaction types (mEPN transition nodes) were defined: protein-protein binding, protein phosphorylation, protein dephosphorylation, protein activation, translocation, protein inhibition, gene expression, catalysis, gene-protein binding and gene repressions (Figure 1B). To reduce the PDS network complexity, degradation was not introduced as a separate reaction type. However, if this regulatory function was specified in the literature (such as, for example, COI1 binding to SCF complex resulting in a degradation of JAZ repressors [37]–[39]), degradation was modelled as a binding reaction.

**Figure 2:**
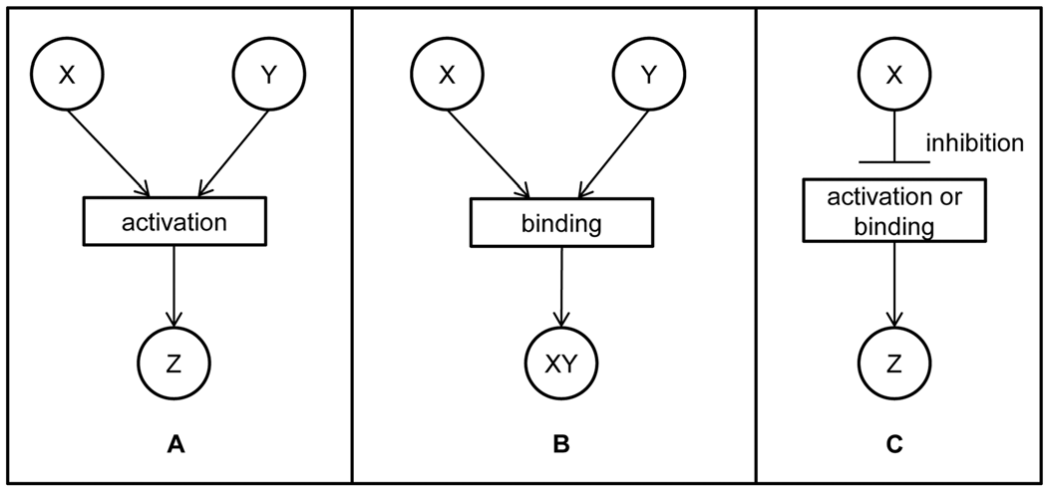
PDS reaction types. There are three groups of reactions. A) *Activation (A)* denotes all the reactions directly involving two components *X* and *Y* in the production of Z, where the concentration Z depends on the concentration of both substrates. B) *Binding (B)* results in the formation of a protein-protein complex or in the binding of a protein to a DNA promoter region to regulate its gene expression. C) *Inhibition (I)* is a process in which one component blocks the performance of another component.

To simplify manual topology construction, the components with similar functions were grouped into families (level 2 in Figure 1A). For example, node named JAZ represents the entire family of JAZ proteins (JAZ1 - JAZ12). The reaction types were also grouped at a higher level of abstraction into three groups (see level 1 in Figure 1B): activation, binding and inhibition. *Activation (A)* denotes all the reactions that follow the principle that, when two components *X* and *Y* are directly involved in the production of *Z*, the concentration of *Z* depends on the concentration of both substrates (Figure 2A). Reactions such as protein activation, phosphorylation of proteins, translocation, gene expression and catalysis are grouped under activation. *Binding (B)* is defined as a close interaction between at least two components resulting in a functional active complex (Figure 2B). Binding is both the formation of a protein-protein complex or the binding of a protein to a DNA promoter region to regulate its gene expression. Binding results in the activation or inhibition of a particular target, gene or protein. Finally, *Inhibition (I)* is defined as a process in which one component blocks the performance of another component (Figure 2C). Inhibition groups all biological reactions such as protein inhibition, gene repression and dephosphorylation of proteins.

The abstraction to the three reaction types (*activation*, *inhibition* and *binding*) at level 1 was necessary to make the PDS model useful for further *in silico* experiments. *Activation* defines processes that activate the next component (or raise its abundance). Even the case of enzymatic reaction where the phosphorylation of a protein results in its deactivation (and thus one would consider it as inhibition) is still classified under *activation* in our work, since when a concentration of a phosphorylated protein increases, then the concentration of a product (which is inactive but phosphorylated protein in this case) also increases. The same holds for the translocation of components or for their degradation. When the active form of the protein is its phosphorylated form, it is this node that is further connected in the model. While in the case when non-phosphorylated form of the protein performs certain function and is thus considered to be the biologically active form of the protein, this node is further connected with the other nodes.

In the manually constructed PDS model topology, the reaction types were encoded according to level 1 shown in Figure 1B and the components were encoded according to level 2 of Figure 1A. The manually constructed PDS network topology consists of three sub-models, each named by the central biological component which is responsible for the defence response of *Arabidopsis thaliana*, i.e., the SA, JA and ET sub-models. The SA sub-model contains 42 biological components and 27 reactions. The JA sub-model contains 33 biological components and 20 biological reactions, whereas the ET sub-model contains 24 biological components and 17 biological reactions. The manual PDS model topology, including additional 4 crosstalk reactions, has in total 99 biological components and 68 reactions. Out of 68 reactions of the manual model, 19 were obtained from KEGG and AraCyc [40], while the rest were found in the PubMed articles. The detailed numbers for individual sub-models forming the manually constructed PDS topology are shown in Table 2 and Table 3. Detailed descriptions of all the components, reactions and corresponding data sources are available in Supporting Information S1.

**Table 2:** Summary of all component types of the manually constructed SA, JA and ET sub-models represented at level 2 of the PDS taxonomy of Figure 1A.

Component types	SA	JA	ET	Total
Small compounds	13	12	6	31
Genes/proteins	27	17	15	59
Complexes	2	4	3	9
All components	42	33	24	99

**Table 3:** Summary of all reaction types of the manually constructed SA, JA and ET sub-models, including the crosstalk connections, represented at level 1 of the PDS taxonomy of Figure 1B.

Reaction types	SA	JA	ET	Crosstalk	Total
Activation	24	16	11	1	52
Binding	1	3	2	0	6
Inhibition	2	1	4	3	10
All reactions	27	20	17	4	68

**Figure 3:**
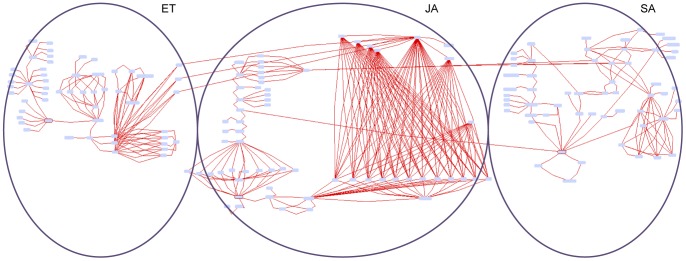
Manually constructed PDS model topology visualised as an edge-labelled graph. This graph, consisting of 175 nodes and 387 edges, is provided in Supporting Information S2 as an interactive graph visualised with the Biomine graph visualisation engine, enabling its closer inspection by zooming into its subparts and rearranging the node and the arc positions in the 2D space. The graph is organised into SA, JA and ET pathways with their crosstalk connections. The node borders of the main pathway components SA, JA and ET are coloured with red.

Modelling the topology at the level of component families (99 components at level 2 of the components taxonomy) is of a manageable size for manually depicting and inspecting the topology graph. However, to allow for in-depth inspection we visualised the PDS model topology at the level of individual components (175 components at level 3). The ultimate result of manual PDS model topology construction is therefore an expanded graph shown in Figure 3. In this edge-labelled graph nodes represent the components and the edges represent the reactions. This is a very complex graph consisting of 175 nodes and 387 reactions, compared to the manually constructed PDS topology consisting of 99 components and 68 reactions.

**Figure 4:**
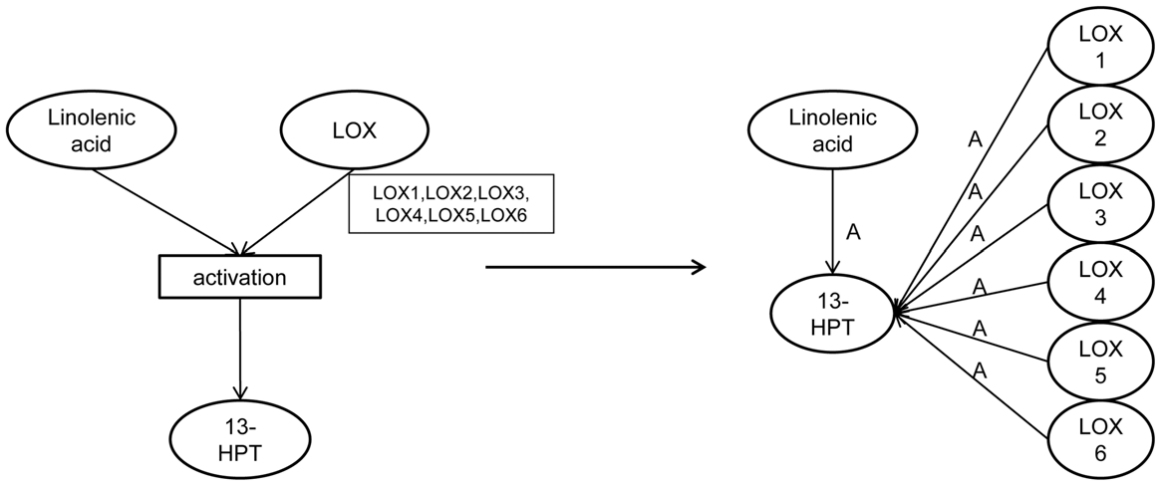
Principle of decomposing families of components by decoupling of reactions. The example shown in this figure presents two conversion types illustrating the transformation from the biological reaction representation into the edge-labelled graph representation. First, the linolenic acid node is connected to the reaction product 13-HPT directly with an arc labelled as A. Second, the decomposing of LOX node is done from the protein family level (level 2) to the single protein level (level 3). The final result of the conversion is a graph with 8 nodes and 7 edges.

The graph of Figure 3 was achieved through automated conversion of the level 2 PDS topology into the expanded level 3 PDS topology, visualised as an edge-labelled graph. This expansion was performed according to the conversion principles outlined in the Materials and Methods section and illustrated in Figure 4. The expanded edge-labelled graph shown in Figure 3 includes 175 components (31 small compounds, 135 genes/proteins and 9 complexes) and 387 reactions (231 activations, 49 bindings, 62 inhibitions and 45 produces reactions from binding reactants to their products). This graph is provided in Supporting Information S2 as an interactive graph visualised with the Biomine graph visualisation engine. Provided that the Java plug-in for the Web browser has been installed and enabled, the reader can open and explore an interactive version of the Figure 3 at: http://ropot.ijs.si/bio3graph/prepareVisualization.php?file=media/supplement/models/Supplement_file_2.bmg.

### Bio3graph Methodology and Implementation

Manual construction of the PDS model topology is a time-consuming process, since only a limited amount of data was gathered in the available biological databases. The study of a large body of literature was therefore necessary in order to build a PDS network structure according to the most recent findings. The proposed Bio3graph methodology was developed with the purpose of automated information extraction from biological literature, aimed at complementing the manually developed PDS topology.

An integral part of this methodology is a domain specific vocabulary that composed of two parts: a list of components and a list of reactions together with their synonyms. The basis for the vocabulary was the list of 175 components and three reaction types defined when building the manual PDS model topology. The components vocabulary consists of their short names, gene identifiers and synonyms, as annotated in TAIR [41] and iHOP [42]. As several components included in the manual PDS model topology are still not fully identified they were labelled as X in the PDS model topology and were not included in the vocabulary. Moreover, in few cases one biological component is represented with two nodes due to its compartmentalisation within the cell. For example, as SA accumulates in both the chloroplast and the cytosol, the manual model node SA which stands for SA in cytosol and node SA-chl which stands for SA in chloroplast are represented by the same component SA. In addition, most of the complexes were not included in the vocabulary except for the SCF complex. Consequently, the list of 153 biological components in the vocabulary (Supporting Information S3) contains fewer components than the vocabulary of 175 components at level 3 of Figure 1A used for manual PDS model topology construction. Furthermore, the vocabulary for the reaction types was developed, containing synonyms for the three reaction types: *activation*, *inhibition* and *binding.* Separate files for each reaction in both the passive and the active verb form are available in Supporting Information S4.

**Figure 5:**
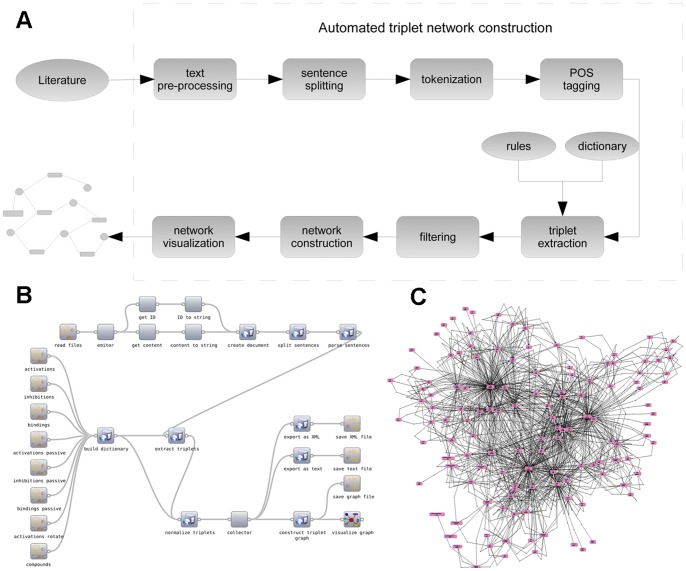
Overview of the Bio3graph methodology, its implementation and a sample output. A) Schematic representation of the Bio3graph methodology. Text processing is performed in a workflow according to the boxes in the schematic diagram resulting in a network of *(component1, reaction, component2)* triplets. B) Bio3graph as a workflow implemented in Orange4WS. C) The triplet network extracted and composed by Bio3graph. The output network (consisting of 129 components and 1,132 reactions) is visualised with the Biomine visualizer and made available in Supporting Information S5.

The Bio3graph methodology consists of a series of text mining, information extraction, graph construction and graph visualisation steps, offering reusability, repeatability, and extension with additional components (Figure 5A). The name of the methodology, Bio3graph, reflects its main functionality: ‘Bio3’ stands for biological triplet extraction and ‘graph’ stands for graph construction from the extracted triplets.

- The first (NLP) part of this methodology (referred to as the *triplet extraction algorithm* below) concerns the extraction of relations in the triplet form *(subject, predicate, object)* thus searching for reactions between components as triplets *(component1, reaction, component2)* from publicly available biological texts by employing the above described manually developed vocabulary. Given the list of components, the algorithm detects *subject* and *object*, while the *predicate* represents the relation between the components as defined in the vocabulary of reaction types. For activation reaction type, an example triplet is *(PAD4, activates, EDS5)*.
- The second part of this methodology concerns graph construction from the extracted triplets, and graph visualisation.

Details of the Bio3graph methodology and its implementation are described in the Materials and Methods section. The methodology is implemented as a workflow in the Orange4WS [43] workflow construction and execution environment (Figure 5B). The input to the Bio3graph workflow is the collection of biological full text articles, obtained through a user-defined keyword-based search of the PubMed Central database, accessible at www.ncbi.nlm.nih.gov/pmc/. The output of the workflow is a network of triplets, automatically extracted from the articles. As an illustration, the network consisting of all triplets extracted from the literature (consisting of 129 components and 1,132 reactions) is shown in Figure 5C and is made available for interactive graph inspection in Supporting Information S5. The triplet extraction algorithm found relations between 129 out of 153 components listed in the vocabulary. If the Java plug-in for the Web browser is installed and enabled, the reader can open and explore an interactive version of the Figure 5C at: http://ropot.ijs.si/bio3graph/prepareVisualization.php?file=media/supplement/models/Supplement_file_5.bmg.

### Evaluation of the Bio3graph Results

The performance of Bio3graph was evaluated on a corpus of 50 full length articles with manually annotated correct triplets. The performance of information extraction is evaluated by calculating the *precision* and *recall* as follows: *Recall = TP/(TP+TN)* and *Precision = TP/(TP+FP)*, where *TP* are the *true positives* (the number of triplets correctly extracted by Bio3graph), *TN* are *true negatives*, *FP* are *false positives*, *TP+TN* is the number of manually identified correct triplets, and *TP+FP* is the number of triplets extracted by Bio3graph regardless if they are correct or not. The results achieved by Bio3graph on the annotated corpus are presented in Table 4 showing average precision of 42.6% and recall of 62.3%. The annotated texts, the simplified vocabulary, together with the Bio3graph results and a detailed summary for each of the 50 papers are available in Supporting Information S6.

**Table 4:** Recall and precision analysis for 50 full-length papers.

Reaction types	TP	TP+TN	TP+FP	Precision (%)	Recall (%)
Activation	142	223	311	45.7	63.7
Inhibition	47	80	134	35.1	58.8
Binding	6	9	13	46.2	66.7
All reactions	195	312	458	42.6	62.3

*Recall = TP/(TP+TN*) and *Precision = TP/(TP+FP)*, where *TP* are the true positives, *TN* the true negatives, and *FP* the false positives. *Recall* is the percentage of the retrieved true positive relations from the whole set of true relations. *Precision* is the percentage of retrieved true positive relations from the whole set of retrieved relations.

Several systems for automated information extraction have already been developed reporting remarkable precision and recall results. Most of them extract the protein-protein interactions from text abstracts or from a filtered text corpus, where only sentences with keywords were considered. For example, the Chilibot system reports a precision from 74.4% for inhibitory relations to 79.1% for the general protein-protein interaction, with a recall of 91.2%. Suiseki has a recall of 70% with the accuracy around 80% for the best defined reactions. The methodology developed by Ono et al. [30] extracted protein-protein interactions for yeast organism with the precision range from 90.2% for the ‘associate’ relation up to 96.1% for the relation ‘interact’ and the recall for the same organism in the range from 80.9% for the ‘associate’ relation up to 89.1% for the ‘interact’ relation. In the same study the recall for extracting a protein-protein interaction in *E. coli* organism ranges from 77.3% for the ‘associate’ relation to 85.2% for the ‘complex’ relation.

The full-length papers have generally a more complex sentence structures than the abstracts. Therefore, when processing full texts both the precision and the recall are lower than in abstract-based relation extraction systems. The only system to which we were able to compare Bio3graph was BioRAT. The BioRAT system achieved a precision of 51.25% and a recall of 43.6%. The average precision of our system is 42.6% which is lower than the precision of BioRAT. On the other hand, recall of Bio3graph is 62.3% which is almost 20% higher than the recall of the BioRAT system. Since Bio3graph was validated on the whole articles and not only the abstracts, we were satisfied with a recall of 62.3%.

In Bio3graph we chose to achieve higher recall at the cost of lower precision, given that the aim of developing Bio3graph was to add to the manually constructed topology the interactions that were missed when manually gathering the information. This means that when used in a real setting, this requires manual reviewing of more false positive triplets, instead of losing some information. As a remark, 62.3% recall does not necessarily mean that we have not detected 37.7% of the interactions, given that the interactions between components are often mentioned more than once in a single paper (in the abstract, results and discussion). It is very likely that if we did not extract one triplet from one part of the article we may still find it in the other parts of the article.

The analysis of non-detected triplets shows that Bio3graph does not cover some sentence constructions. One of these constructions is: “EDS1 protein activates not only EDS5, but also activates SA”. In this sentence there are two triplets *(EDS1, activates, EDS5)* and *(EDS1, activates, SA)*. However, if, for example, EDS1 is not in the vocabulary of compounds, and the other two compounds EDS5 and SA are in the vocabulary, then the algorithm would find the following triplet *(EDS5, activates, SA)*, which is a false positive.

We have also noticed that some triplets were not detected due to incorrect sentence parsing by the GENIA tagger. For example, in the sentence “Expression of NPR1 and defence genes was induced by harpin to higher levels, while only MeJA activated COI1.” the triplet *(MeJA, activates, COI1)* cannot be extracted, because the word “activated” was labelled as a noun phrase instead as a verb phrase.

### PDS Model Topology Extracted by Bio3graph from Biological Literature

Using the following set of keywords: “Arabidopsis thaliana” AND {“defence” OR “defense” OR “ethylene” OR “jasmonate” OR “jasmonic acid” OR “salicylate” OR “salicylic acid” OR “pathogen” OR “virus”}, 9,586 relevant PubMed Central articles were retrieved on April 4, 2011. PubMed Central database was used as it enabled us to gather freely available full text articles and not only their abstracts. These articles were taken as a ground information corpus from which the triplets were extracted. Keywords were selected to obtain the most of the PDS related literature with the emphasis on the JA, ET and SA signalling pathways. Since PubMed Central is a medically oriented database and does not cover some of plant sciences related journals, it is possible that some PDS related articles were not retrieved. Nevertheless, PubMed Central represents the largest source of full text scientific papers and is therefore a relevant basis for our work.

The result of using the Bio3graph triplet extraction algorithm is a set of 1,132 unique triplets, identified from the total of 4,204 extracted triplets. To evaluate the correctness of the extracted triplets, we have manually inspected the sentences from which the triplets were extracted. Since some of the 1,132 triplets appear in several sentences, we have defined the term *correct triplet* in the following way: If the triplet is a true positive in at least one sentence of the whole text corpus, it is considered to be a *correct triplet*. The graph constructed from 377 correct triplets is available in Supporting Information S7. The reader can open and explore an interactive version of the Supporting Information S7 at: http://ropot.ijs.si/bio3graph/prepareVisualization.php?file=media/supplement/models/Supplement_file_7.bmg, if the Java plug-in for the Web browser has been installed and enabled.

Most of the relations found by the triplet extraction algorithm are the ones related to *activation* (out of 1,132 unique triplets in total, 736 are the *activation* reactions between the components). There are fewer *inhibition* relations and very few relations of *binding* type. We have already identified most of these relations when manually constructing the PDS model topology, while some of them are new. Some of the extracted triplets represent direct interactions between the components (i.e., relations between direct neighbours in the graph), while others are indirect (i.e., paths composed of a set of direct relations). A direct interaction is defined as the transduction of the signal between two components without an additional in-between component. For example, the binding of ET to its receptor ETR1 is a direct interaction while the activation of ERF proteins by ET through a signalling cascade is defined as an indirect interaction.

**Figure 6:**
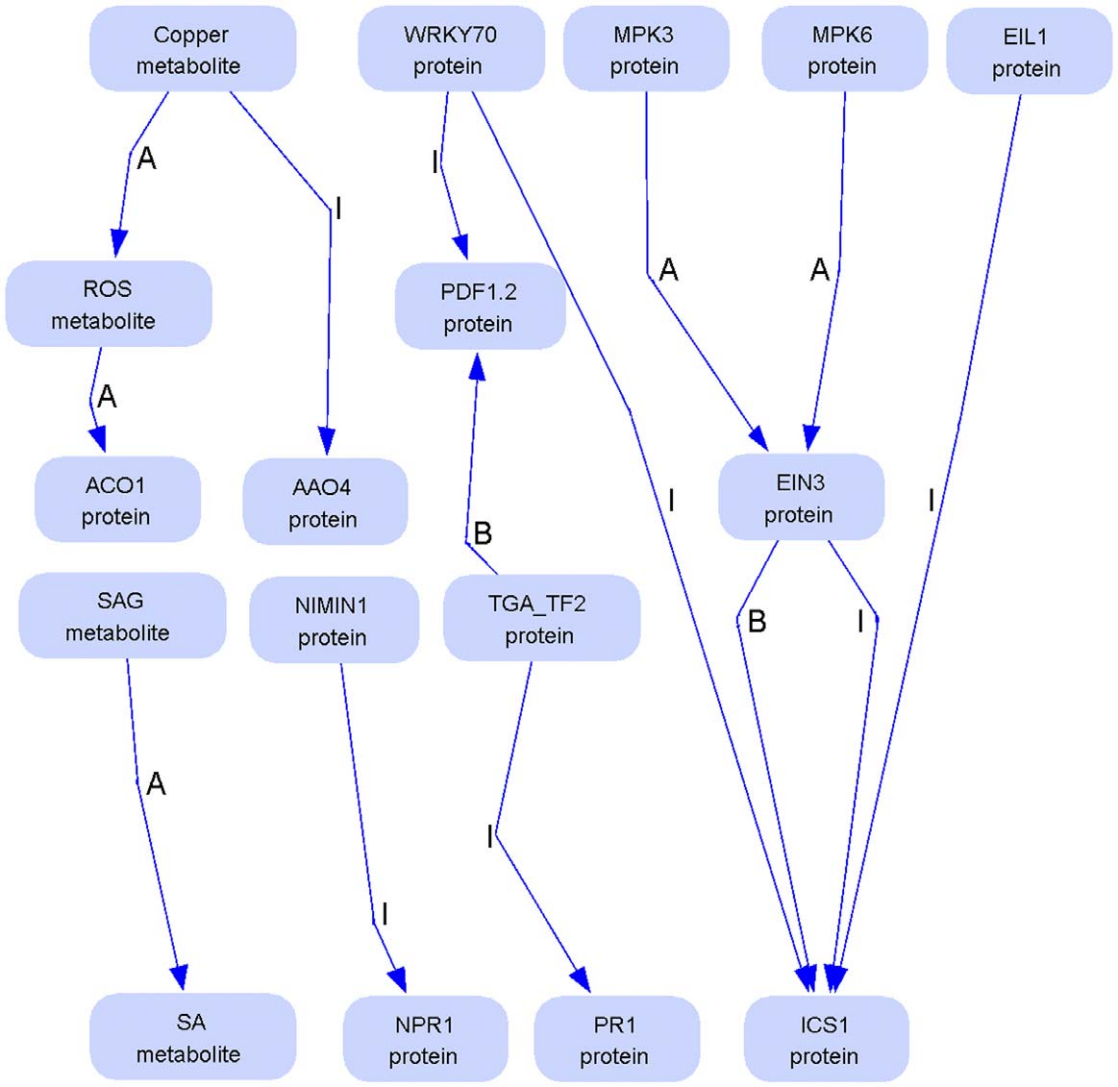
New direct PDS relations extracted from the biological literature. The new direct links result from the Bio3graph processing of 9,586 articles. Bio3graph extracted 14 new direct relations between the components which were not identified in the manually built PDS model topology. Note that two of these triplets are trivial (*SAG_metabolite, activates, SA_metabolite)* and *(NIMIN1_protein, inhibits, NPR1_protein*).

Table 5 gives a summary of the automatically extracted relations between the biological components, emphasizing the numbers of newly discovered direct links (last column of Table 5) discovered by the Bio3graph triplet extraction algorithm. Details of the evaluation for each extracted triplet are provided in Supporting Information S8. The obtained new direct links are visualised in Figure 6, while all the correct new (direct and indirect) links discovered by the triplet extraction algorithm are available in Supporting Information S9. The reader can open and explore an interactive version of the Supporting Information S9 at: http://ropot.ijs.si/bio3graph/prepareVisualization.php?file=media/supplement/models/Supplement_file_9.bmg, if Java plug-in for the Web browser has been installed and enabled.

**Table 5:** Summary of PDS related triplets extracted by the Bio3graph triplet extraction algorithm from 9,586 PubMed Central articles.

Reaction types	Total triplets	Incorrect triplets	Correct triplets	Manual indirect links	Manual direct links	New indirect links	New direct links
Activation	736	446	290	158	41	86	**5**
Inhibition	352	289	63	18	1	37	**7**
Binding	44	20	24	20	2	0	**2**
All reactions	1,132	755	377	196	44	123	**14**

In total, 1,132 triplets were extracted, out of which 377 are correct. Out of these, 14 are newly discovered direct relations and 123 are indirect, while 44 direct and 196 indirect connections were already included in the manual PDS model topology of Figure 3.

Each of the subclasses of correct triplets has its own significance with respect to the PDS model topology. With correct manual direct links we confirmed the applicability of the Bio3graph approach. More importantly, reactions which have not been identified when building the manual PDS model topology (i.e., new direct and indirect links) extend our knowledge on the topic and are therefore of high importance. Indirect relations serve as a database of signal transduction knowledge. Depending on the experimental setup in which these interactions were observed, some of the relations can be redundant. In a biological experiment a hormone can be applied to the plant in order to investigate its effect on the genes of the interest. For example JA can be applied (in the form of MeJA) to the plant and the expression of lipoxygenase (LOX) or oxide synthase (AOS) genes can be monitored in comparison with the plants that have not been pretreated. If both genes show equal increase in the gene expression level, two triplets *(JA, activates, LOX)* and *(JA, activates, AOS)* can show redundancy. If AOS is also activated by LOX, increased level of AOS can be due to the JA-induced activation of LOX and not necessarily due to its activation by JA. In this case a more detailed manual inspection and biological validation has to be performed prior to the incorporation of the links into the PDS model and its simulation.

Using Bio3graph we discovered 14 new direct links, out of which two were known to the biological experts but not included in the manual model as we limited ourselves to the most important elements of plant defence signalling when building the manual model ((*SAG_metabolite, activates, SA_metabolite)* and *(NIMIN1_protein, inhibits, NPR1_protein)).* In the former, only the inverse reaction was included, i.e., (*SA_metabolite, activates, SAG_metabolite), w*hile in the later, the interaction in the manual model was specified as *binding* instead of *inhibition*, which has the same biological function (diminishing the concentration of active NPR1 through binding to NIMIN1). An interesting result is also the identification of components connected with two relations, from which one is a subset of the other. This is the case of ETHYLENE INSENSITIVE 3 (EIN3) and ISOCHORISMATE SYNTHASE 1 (ICS1) where the components are connected with two relations: *binding* (B) and *inhibition* (I) (Figure 6). Biologically this is interpreted as binding of EIN3 to ICS1 causes its inactivation. Biological relevance of the most interesting new direct links is investigated in more detail below.

EIN3 and ETHYLENE INSENSITIVE 3-LIKE1 (EIL1) have been mostly studied as regulators of the ET signalling pathway. In addition to the involvement in the signal transduction of ET-mediated response, Bio3Graph search identified that EIN3 and EIL1 are negative regulators of ICS1. In the paper from which these triplets were extracted it was indeed shown that these two transcription factors inhibit gene expression of ICS1, which is one of the crucial enzymes involved in the SA biosynthesis. The reduction of SA biosynthesis results in a repression SA-mediated signal transduction which is highly-induced upon a pathogen attack [44]. This study was performed on Arabidopsis plants infected with *P. syringae* bacteria. We did not consider this relation when manually building the signalling network topology, as such links between hormone signalling modules are especially difficult for researchers to explicate. Due to automated knowledge extraction, such new ‘out of the box’ thinking results in the discovery of additional knowledge.

Another set of new relations identified by Bio3Graph was related to the WRKY family of transcription factors and Mitogen-activated protein kinases (MAPKs or MPKs). Both are relatively large gene families with very specific functions of individual family members. Therefore they represent a substantial challenge in manual PDS network construction and the information related to PDS was overlooked by the biology experts. Indeed, Bio3Graph identified several missing relations. WRKY70 is known to repress the ISOCHORISMATE SYNTHASE 1, SID2 (also known as ICS1) transcription, although detailed inspection of the manuscript showed that it is still not clear whether it directly binds to the SID2 promoter or not [44]. The WRKY70 transcription factor also suppresses the MeJA-induced expression of PDF1.2 [45] showing its importance in a cross-pathway communication. MAPKs are signal transduction components which play an important role in plant responses to biotic stress. Their performance is cascade-mediated via a complex phosphorelay mechanism. MPK3, MPK4 and MPK6 are the best characterized MAPKs in Arabidopsis [46] [34] and, were thus incorporated in the PDS network. With Bio3graph a new relation was identified in the literature. A modelling approach has suggested an activation reaction between EIN3 and MPK6 [47]. In addition, wet-lab experiments have shown that both MPK3 and MPK6 stabilize EIN3 through phosphorylation of threonine 174 [48]. MPK3 and MPK6 are therefore both positive regulators of EIN3.

Another interesting new relation identified by Bio3Graph is the inhibition of AAO4 by Cu2+. Ibdah et al. [49] have shown that the high concentration of Cu2+ reduces the enzymatic activity of AAO4 for 95%. This fine-tuned activation of AAO4 activity is also an additional reaction revealed by our PDS model topology that could be essential in further kinetic studies.

### The Merged PDS Model Topology

**Figure 7:**
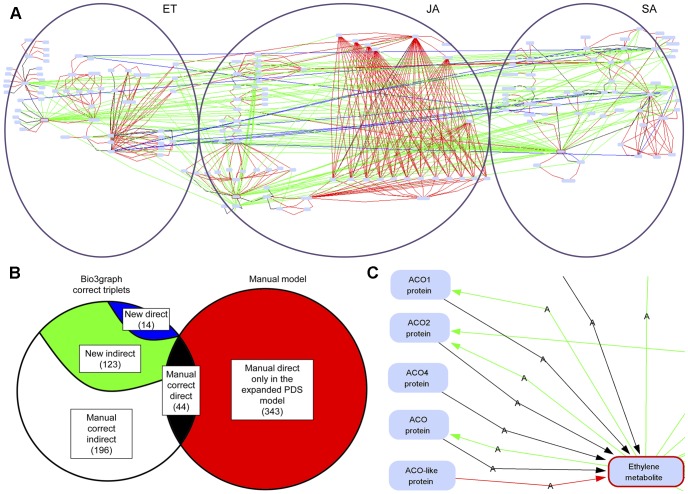
The final PDS model topology constructed by merging the manual and the Bio3graph networks. A) Edge-labelled graph representing the merged model. B) The Venn diagram. The relations in the manual model are all direct and are coloured in red. The intersection between the model relations and the correct triplets extracted from the literature is presented with black colour. From the correct new triplets, the indirect relations are represented with green and the direct ones with blue colour. C) Zoom-in into a part of the merged PDS topology. The links from the manual model are shown in red, while the green coloured relations represent the extracted new indirect links, blue arcs show new direct links and the black arcs show the intersection between the manual model and the correct triplets extracted with Bio3graph.

The manual PDS model topology and the new triplets extracted from the literature were merged into a single graph consisting of 175 components and 524 reactions. This graph, visualised with the Biomine visualisation engine, is shown in Figure 7A. The merged PDS model topology is available for interactive inspection in Supporting Information S10. The reader can open and explore an interactive version of the Figure 7A at: http://ropot.ijs.si/bio3graph/prepareVisualization.php?file=media/supplement/models/Supplement_file_10.bmg, provided that the Java plug-in for the Web browser has been installed and enabled. Bio3graph found 44 direct relations from a total of 387 from the manually built PDS model topology (Figure 7B). A reason for this relatively low overlap is that Bio3graph does not process tables or figure images, out of which the information for the manual PDS model topology was also constructed. Moreover, as Bio3graph processes only one sentence at a time, it does not deal with co-reference. Considering co-reference, which could have further improved the results of triplet extraction, is a possible direction of our further research.

The merged PDS model topology thus represents a faithful representation of current knowledge on a topology of plant defence signalling with the emphasis on plant-virus interaction. More specifically, we have chosen to base our model on resistant interaction between Arabidopsis and virus TCV. In Arabidopsis, the resistance to TCV is mediated by the R protein HRT [49], which subsequently induces the signalling cascade leading to plant defence response which limits viral spread and multiplication. Activation of HRT protein stimulates accumulation of SA [50]. SA in *Arabidopsis thaliana* is synthesized via two pathways both requiring chorismate as a substrate. One pathway goes through a subset of enzymatic reactions initially catalysed by phenylalanine ammonia lyase (PAL) and its homologues (PAL 1,2,3,4). Most of the SA is however synthesized via reaction, catalysed by isochorismate synthase (ICS) and isochorismate pyruvate lyase (IPL) [51]–[52]. Arabidopsis encodes two ICS enzymes, ICS1 and ICS2 [51] [53]. SA accumulation results in the monomerization and the activation of NPR1, which consequently triggers the activation of the SA dependant PR proteins [54]–[55] [2].

SA signalling is fine-tuned with negative and positive feedback loops. A negative feedback loop slows down a signalling process, while the positive feedback loop has a tendency to accelerate it. The final cascade product regulates its own concentration by activating or inhibiting the genes involved in its biosynthesis. NPR1 inhibits the expression of PAD4 and EDS1 [56], two genes involved in the production of SA and consequently, diminishing its own production thus forming a negative feedback loop.

The main biosynthetic pathway for JA is oxylipin pathway, linolenic acid being a substrate for JA biosynthesis [35]. JA can be derivatised to different amino acid conjugates. Jasmonyl-isoleucine (JA-Ile) is the conjugate whose biological activity has been proven [57]. In the presence of JA-Ile, the SCF complex, composed of a SKP1 (S-phase kinase-associated protein 1), cullin, and a RING finger protein (RBX1/HRT1/ROC1), binds to F-box protein Coronatine insensitive1 (COI1). SCF^COI1^ ubiquitine ligase binds to JAZ and presumably ubiquitinases it [35] [58]–[59] [37]. When ubiquitinated JAZ repressors are targeted for degradation in 26S proteasome, they result in the de-repression of the transcription factors such as the MYC2 and other beta helix-loop-helix transcription factors [60] which activate JA-dependant PR gene expression [2].

L-methionine is transformed by S-adenosyl-L-methionine (SAM), 1-amino-cyclopropane-1-carboxylate synthase (ACS) and ACC oxidase (ACO), to form a gaseous hormone ET [61]. When synthesized, ET binds to its receptors. There are five membrane-located receptors identified in Arabidopsis (ETR1, ETR2, EIN4, ERS1 and ERS2) [48] [62]. Binding of ET to its receptor leads to CTR1 deactivation, which finally results in downstream activation of EIN3/EIL1/EIL2 transcription factors [48] [63]. CTR1 levels are also regulated by ubiquitination and 26S proteasome degradation via EBF1/EBF2 - Skp- Cullin-F-box (SCF) E3 ligase complex [62]. The concentration has to be well regulated, since they are the crucial positive regulators of ET signalling.

SA, JA and ET pathways do not function independently, but are rather interconnected by agonistic and antagonistic interactions to fine-tune the plant defence response. These regulations are very complex and often more than one component is involved in the signal transduction [61] [13] [64]. When Bio3graph was applied to enhance the manually built PDS model topology, most of the newly-found relations were characterized as ‘indirect’. Most of them indicate a cross-talk between the sub-pathways or a feed-back regulation of the crucial components in the model. However, some new Bio3graph links are direct. A cross-talk link connects ET and SA sub-pathways: MPK3 and MPK6 activate ET signalling pathway transcription factor EIN3 which negatively regulates SA biosynthesis trough binding to ICS1 (Figure 6). Using dynamic modelling approaches the SA concentration changes can be simulated in different model topologies. If we consider only the manually built model only NPR1 and MPK4 affect negatively the SA concentration. Removing these two proteins from the model by *in silico* knock-out results in an infinite rise of SA. We assume that in a real biological experiment with NPR1/MPK4 double mutant the SA levels would drop thus implying the other negative regulators of SA biosynthesis are involved. Adding the cross-talk link with ET sub-pathway found by Bio3graph could improve the model to more accurately predict SA concentration changes in such knock-out plants.

Bio3graph is used also for extracting detailed information about certain protein family. Several enzymes that are members of the same family can be involved in one biological reaction. For example, according to AraCyc there are five ACC oxidases (ACO1, ACO2, ACO4, ACO and ACO-like) catalysing the last step of biosynthesis of ET [65]. Evidence for their 1-amino-cyclopropane-1-carboxylic acid oxidase activity can be obtained either from the experimental data or from a computational prediction, which is usually sequence-based. With Bio3graph we were able to extract the data for each of the family members, compare the experiments and evaluate their importance for the model. In Figure 7C one can see that Bio3graph identified all ACO family members, apart from the ACO-like, activating ET production. When manually checking the papers we established which relations represent biochemical knowledge and which interactions rely only on sequence homology data. Such detailed information can be used in further dynamic modelling experiments.

As shown in Figure 7C five proteins belonging to ACO protein family have manually assigned activation links in our model. Triplet extraction tool Bio3graph has confirmed four out of five activation links. The activation link between the ACO-like and ET originates from the manual construction process of the structure (after expanding from level 2 to level 3– see Figure 1A) and has not been confirmed by Bio3graph. ACO-like therefore either has a different function than other members of this protein family or it was not explicitly determined as ET biosynthetic enzyme in the literature surveyed by Bio3graph. To determine the real function of ACO-like biological experiments should be conducted using methods that reduce or increase the expression of genes encoding ACO-like. For example, reducing ET concentration in Arabidopsis knock-out plants would confirm the involvement of ACO-like in ET biosynthesis.

Indirect links found by Bio3graph can also guide researchers to form new hypotheses and perform experiments guided by model predictions. For example, indirect link *(SA, activates, EDS1)* (see Supporting Information S10 for a detailed view) means that it is not precisely known whether SA directly activates EDS1 or it activates some of the biosynthesis components upstream from EDS1 which results also in activation of EDS1. The exact nature of such activation can be checked and tested in the laboratory experiments. Nevertheless, this link provides a first clue about the existence of a positive feedback loop in the SA pathway.

### Conclusion

This paper presents the PDS network topology, developed by combining a manual and an automated topology construction approach. This topology is, to our knowledge, the first of this scale, comprising most of the information available on interactions between the components of SA, JA and ET pathways.

The manual PDS topology construction approach was upgraded with a new Bio3graph approach which combines semi-automated extraction of new relations between the biological components with a graph construction and visualisation approach. The main advantage of our method is its effectiveness and reusability. We have implemented the basic vocabulary and accomplished valuable results in terms of precision and recall. Its overall design allows the output of Bio3graph to be easily transferred to standard systems biology modelling formalisms.

The presented approach may appear labour-intensive, as we have started by manually building the initial network structure, which required substantial human expert involvement through time-consuming acquisition and analysis of available information in the databases and literature. Note, however, that the developed Bio3graph information extraction tool can be used from scratch, without first manually building the network structure. Such an approach would be less labour-intensive, as automatic information extraction algorithms examine papers on behalf of the researchers and save experts’ time from examining the full-length documents. These algorithms aim to be research assistant tools for human experts, but they can never replace human expertise. Nevertheless, these tools always require a certain amount of effort for the manual setup of the tool (vocabulary definition like in our work, or template definition like in BioRAT [32]). Also, when the expert wants to apply the information extraction algorithm to a new field of interest, after automatic generation of results, the expert still has to check all the results manually to assure their correctness since there is no algorithm with absolute accuracy.

The presented approach may also appear error-prone, achieving 62.3% recall and 42.6% precision when applied on full-length articles. However, especially in the natural language processing field, the precision and recall of a specific algorithm can vary a lot depending on the text corpus that is processed [32]. For example, as Corney et al. report [32], the BioRAT system has a recall of 20.31% with 55.07% precision when applied to scientific abstracts, while the same algorithm achieves 43.6% recall and 51.25% precision on full-length papers. We have achieved comparable results, but have traded-off lower precision for a higher recall, as high recall is needed to extract as many new relations between biological components as possible. The advantage of our system is its public availability and simple reuse, as Bio3graph is available as a repeatable workflow in the Orange4WS publicly available data mining environment. Most importantly, by employing a different vocabulary, Bio3graph can be reused to extract the network structure of other biological mechanisms.

Some graphical interfaces allow manual removal of the incorrect connections from the graph [66]. Similarly, in future work we plan to add this feature to upgrade the BioMine engine [23]. We also plan to use the developed Bio3Graph approach to upgrade the BioMine engine with an automated step of triplet extraction from literature and automated construction of the initial BioMine network, when building the BioMine database for a new domain such as plant biology, for which this resource has not yet been developed. Moreover, the PDS model topology will serve as a baseline for further research in the area of plant-pathogen interactions. It can be easily transformed into formalisms enabling dynamic modelling, and through simulations and predictions identification of critical components in plant defence signalling can be achieved more efficiently.

We expect that the Bio3graph tool will be used for building topologies of other biological processes as well. With the possibility to manually adjust the vocabulary of components and their interactions, the Bio3graph approach can be used for in-depth literature exploration on a selected topic. The same approach can be also used for incremental topology updating based on triplet extraction from recent literature, as it is time consuming and sometimes impossible for the scientists to track the newest findings in the literature. Even though this approach can not completely substitute the experts, it can significantly speed up knowledge elicitation from the literature. The presented results indicate the usefulness of the proposed approach but also the necessity to further improve the quality of information extraction.

## Materials and Methods

### Manual Model Topology Construction

The literature inspected for the manual model construction was selected by the domain experts. We have focused on scientific papers related to the PDS field. Ideally, a citation in one review paper or in two scientific papers was required for the inclusion of information into the pathway diagram. If the information was available in one publication only, we critically assessed the publication quality (e.g. high impact factor, author’s relevance in the field) before incorporating this information into the model. The information about the biosynthetic pathways and other available data was acquired from different databases such as KEGG [18] and TAIR [41]. KEGG was used as a backbone for building the metabolic pathways, the biosynthesis of the hormones and the main reactions involved in this processes. Additional reactions and genes involved were implemented according to the Aracyc database. TAIR provided gene information, and synonym names were acquired from iHOP [42] and TAIR [41]. A list of all the topology interactions was compiled, including the details of the information sources, and is available in Supporting Information S1.

Members of component families were selected according to their function. The family members were joined under a common family name if more than one component (family member) could be involved in the listed reactions. The genes involved in hormone biosynthesis are usually well described and implemented in the reaction scheme of different databases such as plant metabolic network. Therefore the component family members of biosynthetic pathways were defined as in these databases. The other relations were manually acquired either from the studied literature (such as ET receptors) or from the TAIR database.

To avoid visual overload, all the components in the PDS model topology of Figure 3 are represented with the same shape and colour, specifying the component type only with text in the graph node. The reaction types (edges) in the expanded topology are represented by the first capital letters of their names: A (*activation*), I (*inhibition*), B (*binding*), P (*produces*).

### Principled Conversion of the PDS Topology to the Edge-labelled Graph Representation

**Figure 8:**
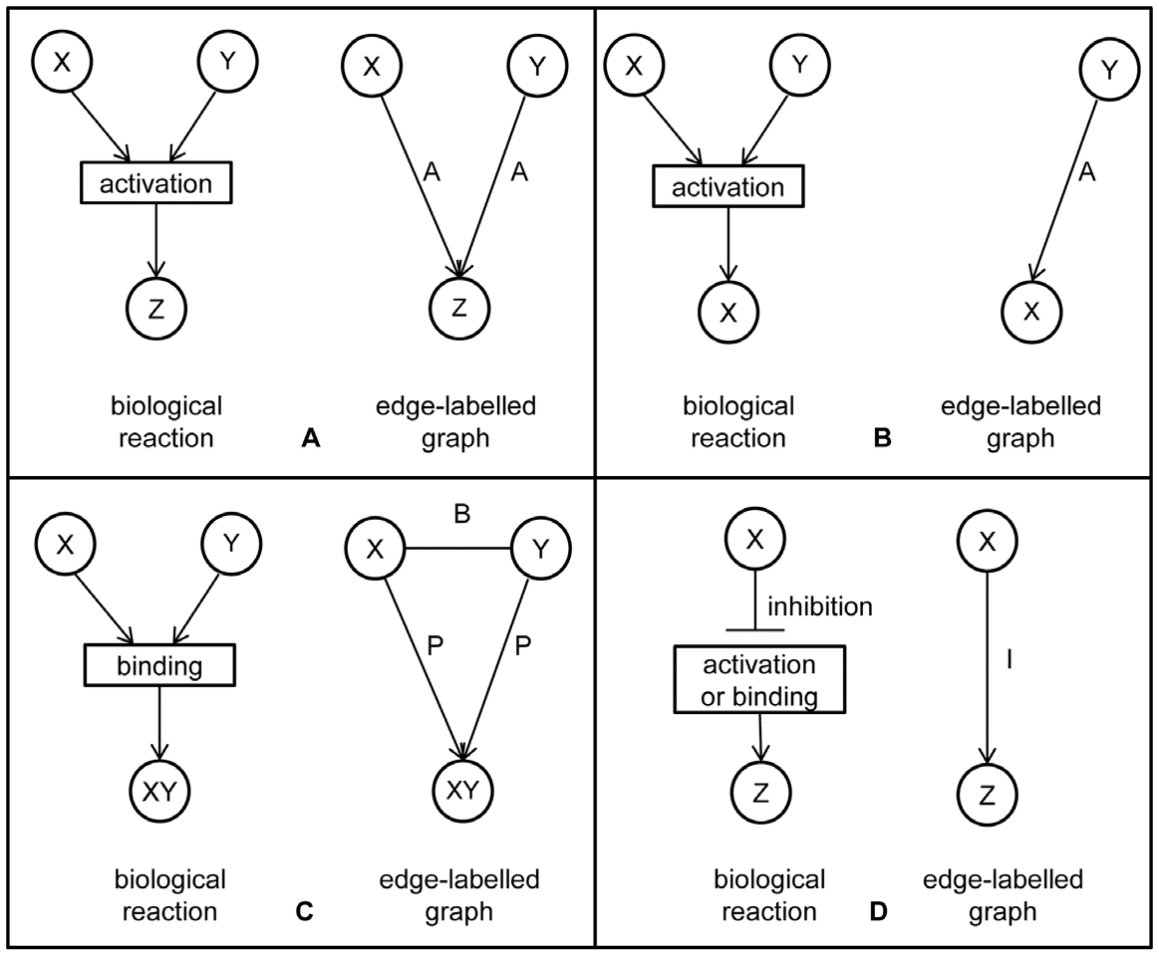
The principles of conversion to the edge-labelled graph format. A) *Activation* reaction (labelled A) reaction between two components is transformed into the graph with arcs between the reactant and the product node. B) *Activation* (labelled A) on a transcription level is a special type of *activation*, when Y induces the activation of gene X to produce protein X. In this case we omit the gene transcription level when transforming the level 2 topology to the edge-labelled graph. C) *Binding* (labelled B) relation between two reactant nodes X and Y is transformed into a *B* relation between the reactants and an additional relation *produces* (labelled as P) between the reactant and the product. The latter is introduced to represent the binding of proteins into complexes. Binding is a binary relation, consisting of a bidirectional edge; in graph visualisation, the arrows are omitted. D) *Inhibition* (labelled I) is the blocking of the activation or binding reaction between components by a third component X, resulting in reduced production of product Z.

As the triplet extraction method for automatically extracting knowledge from the literature results in a graph consisting of *(component1, reaction, component2)* triplets, and as our aim was to enhance the manually constructed PDS model topology with automatically extracted triplets, we had to convert the manually constructed PDS model topology into the compatible format. This is the edge-labelled directed graph format, where nodes represent the components and edges represent the reactions. This conversion was performed by decoupling the reaction of two components resulting in a joint product, into two relations, one for each component connected to the product. Details of this conversion are presented in Figure 8.

In addition, the decomposition of the nodes which represent a component family into individual nodes was needed in order to ensure the compatibility of the manually constructed topology and the automatically extracted graph consisting of *(component1, reaction, component2)* triplets. Reaction decoupling into separate relations is illustrated in Figure 4. The members of the family were selected from the plant databases, Aracyc and TAIR and additional topic-related papers in the way that most of the functional analogues were listed. The list of the PDS model topology components for which decoupling was performed is available in Supporting Information S11.

### Bio3graph Methodology

For natural language processing we employed functions from the Natural Language Toolkit (NLTK) library [67]. Additionally, the GENIA tagger [68] for biological domains was used to perform part-of-speech tagging and shallow parsing. The data were extracted from PubMed Central using web-service enabled access. Parts of the Bio3graph methodology, presented in Figure 5, are described in more detail below.

#### Text pre-processing

The full texts of scientific articles for biology domain are accessible in the publicly-available databases, such as PubMed Central. The journal articles in the form of raw text need to be pre-processed. For example, in order to avoid a false detection of et (ethylene) component by the algorithm, the phrase “et al.” was transformed into “ETAL.”.

#### Sentence splitting

When the raw text is obtained with the previous module, the sentences are separated into lines. This step is necessary because the input into the Genia tagger module requires one sentence per line in the text file.

#### Tokenization

This is the process of splitting a sentence into words, phrases or other meaningful elements referred to as tokens. Tokenization is performed with the Genia tokenizer [69]. The outputs of the tokenization process are tokens that are used for POS tagging, i.e., shallow parsing of the sentence.

#### POS tagging and chunking

Part of speech (POS) tagging is the process of labelling each word in a sentence as a noun, verb, adjective, adverb, etc. Chunking is the labelling of the sentences into the syntactically-correlated groups of words such as noun phrase (NP) and verb phrase (VP). For the purpose of POS tagging and chunking of biological texts we used the GENIA tagger. The output from the GENIA tagger are the chunks of the words, such as Noun Phrase (NP), Verb Phrase (VP), etc. These output chunk labels are the phrase levels according to the Bracketing Guidelines for Treebank II Style Penn Treebank Project (see publicly accessible phrase level types at http://bulba.sdsu.edu/jeanette/thesis/PennTags.html). The sentence labelled with the chunk labels is the input to the triplet extraction module.

#### Triplet extraction

The aim of the triplet extraction algorithm is to find the triplets in the form of *(subject, predicate, object)*. If the *predicate* is in active form, the *subject* is the part of the noun phrase (NP) before, and the *object* is the part of the noun phrase (NP) after the *predicate*. The opposite holds for the passive form of *predicate*. *Predicate* is either one word that belongs to the verb phrase (VP), or it is a multi-word phrase, partially belonging to the VP. The output from the triplet extractor is the triplet list together with the sentence from which the triplet was extracted and the article PubMed Central ID number. Triplet extraction is performed by employing rules with the help of a manually developed vocabulary of components and reactions:

- Components vocabulary contains a list of all components of the manually developed PDS model topology.
- Reactions vocabulary consists of three different types of reactions (activation, inhibition and binding) together with their synonyms and synonym phrases. For example, the activation in Figure 1B has induces as a synonym, but also the whole phrase shows increased levels in the presence of.

We have categorised triplet extraction rules into three categories: a rule for one-word predicates, a rule for multi-word predicates and a rule for swap. We describe briefly each of them.

**Figure 9:**
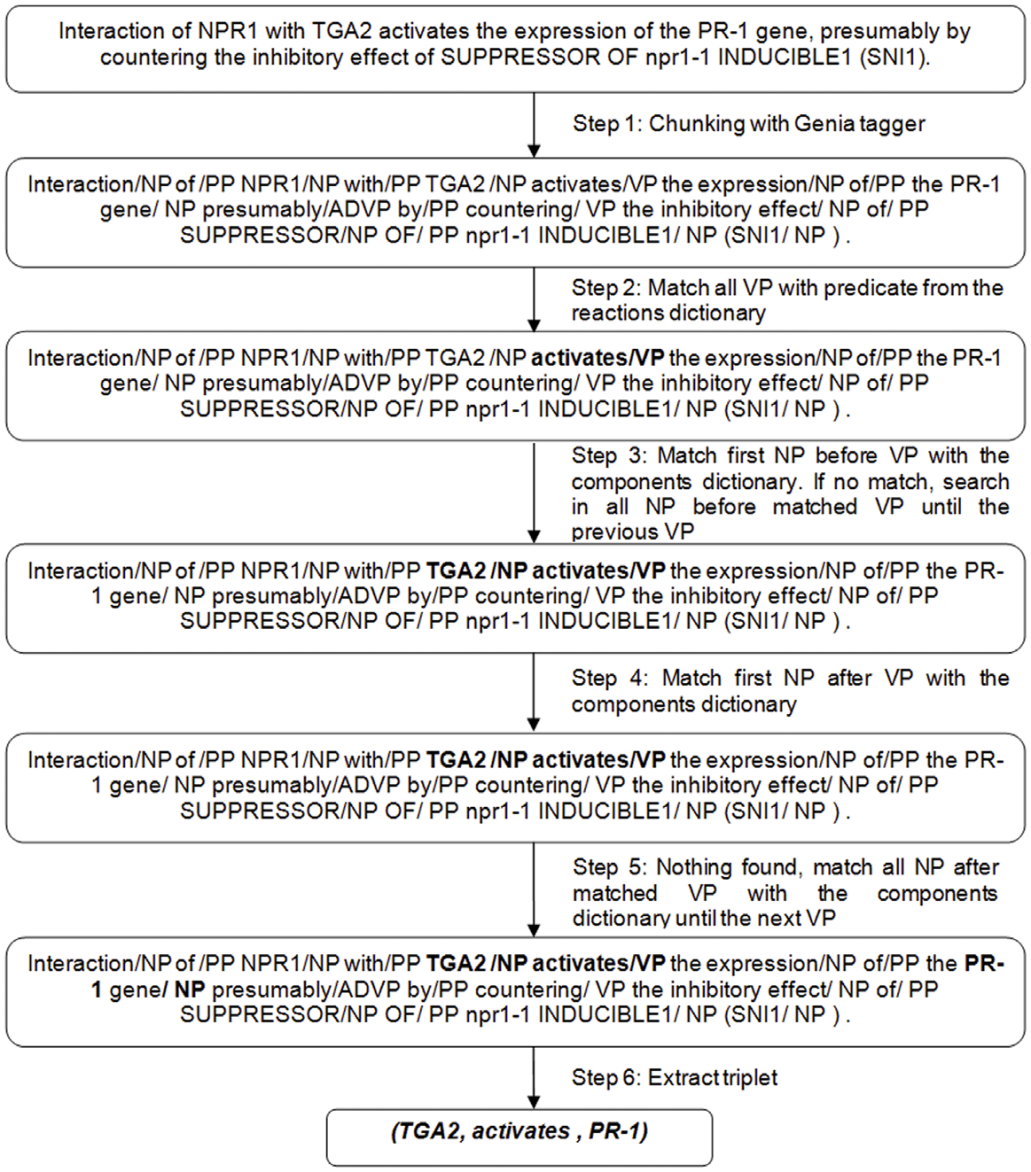
Illustration of the triplet extraction. We show a part of the flow from input of POS tagging box from Figure 5 until output of triplet extraction box of the same figure. The input to the Genia POS tagger is previously pre-processed sentence. After the shallow parsing with Genia POS tagger, the algorithm performs the step 2. The final output from the triplet extraction part of Bio3graph approach is a triplet in the form *(subject, predicate, object)* which will be then transformed and visualised as an edge-labelled graph with the Biomine visualiser.

- One-word predicate rule deals with the *predicate* that is only one word, such as: *activates*, *stimulates*, *reduces*, etc. The algorithm for triplet extraction of this rule is shown in Figure 9. After the sentence is chunked into chunk tags with the Genia tagger, we first compare all VPs with the reactions vocabulary (step 2 in Figure 9). If at least one match is positive, we define it as a *predicate*. Next, we search for the *subject* and the *object* in the Noun Phrases before and after the *predicate*. The comparison between the NP before the detected VP and the components vocabulary (step 3 in Figure 9) is performed first. This match provides the *subject*. Next, the match is done between the NP, after detected VP, and the components vocabulary (step 4 in Figure 9). If the match is negative, the matching continues between the next NPs and the components vocabulary until the next VP in the sentence (step 5 in Figure 9). If the match is positive, the *object* is detected and the triplet is finally extracted (step 6 in Figure 9).
- Multi-word predicate rule addresses the search for triplets when the *predicate* is a phrase with more words, such as *is a positive regulator*, *is suppressor of*, *shows increased accumulation*, etc. The *subject*, the *predicate* and the *object* are searched in a similar way as in the Rule for One-Word Predicate.
- Rule for swap is applied if the *predicate* is in the passive form or if the *predicate* matches the activation_rotate vocabulary file (see Supporting Information S4)**.** In these cases the places of *subject* and *object* are swapped.

#### Filtering

Filtering of extracted triplets is performed in order to reduce the false negatives. The filtering box removes the triplets from the extracted ones if they belong to any of the following categories:

- Triplets with the same *subject* and *object*, for example: *(EDS1, activates, EDS1)*.
- Triplets that are extracted from ‘hypothetical’ sentences, such as: “It was studied whether EDS1 protein possibly activates EDS5 gene”. The following set of ‘hypothetical’ words was defined: *possibly*, *whether*, *to determine*, *to investigate*, *to study*, *it was postulated*, *it was hypothesized*. If these ‘hypothetical’ words were detected in the sentence, the triplet was filtered out. Also, if the words like: *may*, *might*, *can*, *could*, *would* were detected in the VP of the *predicate*, the sentence is considered ‘hypothetical’ and the triplet is filtered out.
- Triplets extracted from the sentences related to mutant plants. A set of ‘mutant plants’ words was predefined, such as: *plant*, *mutant*, *line*. If these were detected in the NP of the s*ubject* or *object*, the triplet was filtered out.
- Too ‘general’ triplets. An example is the following sentence: “The activation of Salicylic acid pathway increases the activity of Jasmonic acid pathway”. Triplet *(SA, activates, JA)* would be extracted from this example sentence. However this triplet is considered to be too ‘general’ since it addresses not only one specific component, but the whole pathway. For this reason, the set of ‘general’ words was also defined for filtering: *pathway*, *signalling*, *synthesis*, *biosynthesis*, *response*, *activator*, *inhibitor* and *producer*. If some of these ‘general’ words were in the same NP of the *subject* or the *object*, the triplet was filtered out.
- Negation triplet. If the VP contains the words *not* or *n’t* the triplet is filtered out. Note that processing of ‘contradictory triplets’ is done by filtering out these negation words.

#### Network topology construction and visualization

Triplets *T* = {*(subject, predicate, object), where subject*, *object* ∈ *Components*, *predicate* ∈ *Reactions*} obtained from the manual model or through Bio3graph are used to construct an edge-labelled directed graph *G = (V, A),* where the set of vertices *V* is the set of all *Components*, and the set of arcs *A* is a set of all *Reactions*. The weights are not assigned to arcs but in general weights can be used to reflect the reliability of the extracted triplet. Note that the graph is not necessarily connected and it does not contain any isolated vertices.

Since the extracted structure can contain a very large number of vertices and many unconnected components it is important to use scalable network visualisation methods, e.g., Barnes Hut n-body simulation [70]. We have employed a freely available platform independent network visualisation component provided by the Biomine system [23] which implements a variant of the force-directed layouting algorithm, and allows for the visualisation and interactive exploration of reasonably large graphs. For example, a picture of a triplet network consisting of 175 vertices and 524 edges as drawn by the Biomine visualisation engine is shown in Figure 7A.

### Bio3graph Tool: its Implementation and Availability

This section discusses the implementation of the Bio3graph methodology and the availability of the Bio3graph tool. We have implemented the Bio3graph methodology in a general framework which is modular and extensible, and provides functionalities at three different levels of generality. The first level provides classes for core data structures such as *Corpus*, *Document*, and *Triplet*, and the related low-level language processing functions such as sentence splitting, tokenization, tagging, and parsing. The second level contains the triplet extraction algorithm, its custom vocabulary data structure, and various utility functions some of which are algorithm-specific. The third level provides post-processing such as normalization and filtering, and exporting of the results as text, XML, graph and other formats.

Our framework is implemented using the Python programming language, and relies on the publicly available Natural Language Toolkit (NLTK) [67] software for natural language processing, and the GENIA tagger [68]. NLTK is a native Python suite of libraries and programs for natural language processing while the GENIA tagger provides part-of-speech tagging, shallow parsing, and named entity recognition for biomedical texts. In order to enable access to the GENIA tagger from the Python language environment we implemented a wrapper which turns the standalone tagger program into a Python library, thus allowing an easy integration with the rest of the framework. In addition, our framework also integrates the Biomine tool [23] for graph construction and visualisation, which enables interactive graph visualization including zoom-in and zoom-out, as well as the relocation of the graph vertices and arcs.

In the triplet extraction workflow, the NLTK library provides sentence splitting [71] while the GENIA tagger is used for tokenization, POS tagging and shallow parsing (chunking) thus forming the backbone of our implementation. Because of the modular structure, the existing software libraries performing various language processing steps can be easily integrated.

In order to provide an easy, system and software independent access to our triplet extraction tool, we have developed a collection of web services that expose the relevant functions of the framework. These services were implemented using the Orange4WS [43] server tools as stateless SOAP web services. Currently, the web service description document (WSDL) and its related XML schema define three data structures (document, dictionary and triplet) and nine functions: *create_document*, *create_dictionary*, *split_sentences*, *parse_sentences*, *extract_triplets*, *normalize_triplets*, *construct_triplet_graph*, *triplets_to_XML* and *triplets_to_text*.

The Bio3graph workflow, shown in Figure 5B, works as follows. First, a dictionary is created by calling the *create_dictionary* function which builds the dictionary structure according to the XML schema from the provided text files specifying the reactions and the components. Then, each loaded document is sent to the triplet extraction workflow. It consists of the following parts: creation of the document structure from raw text data, sentence splitting, shallow sentence parsing, triplet extraction algorithm and triplet normalization (removal of duplicates, change of order in the case of passive *predicate*, and base word formatting). The resulting list of triplets is then saved into a text and XML file, transformed into a graph by Biomine and finally visualised in the Biomine interactive graph visualiser. Note, however, that the triplet extraction workflow is enclosed between the *emitor* and *collector* components, provided by Orange4WS, which enable simple, unconditional iterations. The emitor component emits elements of the input iterable object, one at a time, while the collector collects the incoming elements into a list. This allows for extracting triplets not only from a single document but from the whole corpus. The Bio3graph tool is publicly available at http://ropot.ijs.si/bio3graph/.

## Supporting Information

Supporting Information S1 The summary of all relations in the manual model. This summary is grouped into four sheets named with the sub-models names: SA, JA, ET and the forth one, crosstalk. Table structure is the same in every sheet. In the first column the interaction between two biological components is given in the form of biological reaction representation, with the following structure: (reactant1+ reactant2 reaction product). For example: *protein_MYC2 + gene_THI2.1JR1VSP1CLH1 activates protein_THI2.1JR1VSP1CLH1*. In the second column the interaction between the two biological components is converted in the format of the edge-labelled graph with the following structure: (reactant, product, reaction abbreviation). For example: *protein_MYC2 protein_THI2.1JR1VSP1CLH1 A*. In both of these columns the biological components are represented on the level of the family nodes. In the third column the relations after decomposition of the family nodes are shown also in the edge-labelled graph format. In the last forth column the source of information, related to the particular interaction, is specified.
(XLS)

Supporting Information S2 A manual expanded graph file represented at the single component level. The visualisation of this file into a graph in an interactive way is possible with the Biomine visualizer file *bmvis.jar*. If a user does not have installed Java software package, he should install it. It is available for download at: http://java.com/en/download/index.jsp. The *bmvis.jar* file can be downloaded from the link: http://www.cs.helsinki.fi/u/phintsan/bmvis.jar and it should be located in the same folder as the Supporting Information S2. To perform visualization of the file one should do the following: 1. Open Command Prompt window 2. Change your path into the folder where the Supporting Information S2 is located. 3. Type into the Command Prompt following: “<absolute path of java.jar file >\java” –jar bmvis.jar “Supporting Information S2.bmg” An example is a following line: C:\Users\Dragana\Desktop\ SUPPLEMENT>“C:\Program Files (x86)\Java\jre1.6.0_22\bin\java” -jar bmvis.jar ” Supporting Information S2.bmg" A user should pay attention to the spaces between words in the example line above and apply them in the same way when visualising the graph. In case a warning message is displayed the user should click OK and the graph will be visualized. Note that this visualisation procedure applies only for the Windows platform.
(BMG)

Supporting Information S3 Vocabulary of the biological components used by Bio3graph tool. In this vocabulary every row represents one component with its synonyms separated by comma. The first name in the row represents the biological component name that is also visualized in the graph nodes.
(TXT)

Supporting Information S4 Vocabulary of the biological reactions used by Bio3graph tool. This vocabulary contains in total 6 files with synonyms for three types of reactions: activation, binding and inhibition in both active and passive form.
(RAR)

Supporting Information S5 The graph file with correct and incorrect triplets found by Bio3graph. The triplet extraction was performed on the set of the 9,586 articles resulting in a file which can be visualized with Biomine visualizer in the same way as the Supporting Information S2.
(BMG)

Supporting Information S6 Materials and results from the precision and recall experimental Bio3graph evaluation. Since the evaluation was performed with the simplified dictionary we supply this simplified version in a separate folder. Next, there are also 50 raw text articles in a folder *txt_files*, which we have used for labelling the triplets to obtain ground truth dataset. Also, uploaded is the Ground truth file, named *GROUND_TRUTH_manually_annotated_triplets.doc* where each triplet from the 50 articles is coloured in different colours depending whether the Predicate is *activates*, *inhibits* or *binds*. Also, the results from Bio3graph are provided in *Bio3Ex_triplets.doc* file. Finally the file *Triplets_summary.xls* represents the summary of all the triplets manually annotated and found by Bio3graph for each article separately.
(RAR)

Supporting Information S7 The graph file with only correct triplets, extracted by Bio3graph. This network (consisting of 107 components and 377 reactions) can be visualized with Biomine visualizer in the same way as the Supporting Information S2.
(BMG)

Supporting Information S8 The summary of triplets, extracted by Bio3graph tool from the 9,586 articles. In the first column of this file the found triplet is represented in the edge-labelled graph presentation way that can be visualized with the Biomine graph visualizer. The names of the Subject and Object name are converted with the first synonym name from the component vocabulary (see the Supporting Information S3). The Predicate name is transformed to the first letter of the reaction type that it belongs to: A from *activates*, B from *binds* and I *inhibits*. The second column represents the same triplet in a form of (Subject, Predicate, Object) with the same normalized names. In the third column are the triplets with the subject, predicate and object names as they are found in the sentence originally. The next columns provide information whether the triplet is correct, new and direct with respect to our manually constructed PDR topology. The last columns contain the PubMed Central ID of the article, publishing year and the sentences from where the triplet is extracted.
(XLS)

Supporting Information S9 The graph file of the newly found triplets. These new direct links are blue coloured and new indirect green coloured. The network consists of 63 components and 137 reactions and can be visualized with Biomine visualizer in the same way as the Supporting Information S2.
(BMG)

Supporting Information S10 The graph file of the merged PDR topology. This topology contains both manual and new triplets from the literature. The arcs from the manual model are all direct and are coloured in red. The intersection between the manual model arcs and the correct triplets extracted from the literature is represented with black coloured arcs. Further, from the correct new triplets, the indirect connections are represented with green and the direct ones with blue colour. The file can be visualized with Biomine visualizer in the same way as the Supporting Information S2.
(BMG)

Supporting Information S11 The levels of the biological component abstraction. The first column in the table represents the family name of the biological component. The second column contains all the single members of the family that are considered important for the plant defence. The third column contains their unique ID numbers from the TAIR database while in the fourth column are all the synonyms for the component names.
(XLS)
